# The link between emotion regulation and size estimation of spiders pictures among women with fear of spiders

**DOI:** 10.3389/fpsyg.2022.1053381

**Published:** 2022-12-23

**Authors:** Yahel Dror Ben-Baruch, Tali Leibovich-Raveh, Noga Cohen

**Affiliations:** ^1^Department of Special Education, University of Haifa, Haifa, Israel; ^2^Department of Mathematics Education, University of Haifa, Haifa, Israel; ^3^The Edmond J. Safra Brain Research Center for the Study of Learning Disabilities, University of Haifa, Haifa, Israel

**Keywords:** size bias, spider, cognitive control, reappraisal, suppression

## Abstract

**Introduction:**

Fear is associated with perceptual biases. People who are afraid of spiders perceive spiders as larger than people without this fear. It is yet unclear, however, whether this effect can be influenced by using implicit (non-deliberate) emotion regulation (ER) processes and explicit (deliberate) ER strategies, such as reappraisal and suppression.

**Method:**

This study examined the link between implicit and explicit ER and size estimation among women afraid of spiders. After performing an implicit ER (cognitive control) task, participants rated the size and valence of spiders, wasps and butterflies shown in pictures. Participants’ tendency to use reappraisal and suppression was assessed using the Emotion Regulation Questionnaire.

**Results:**

Results showed no effect of implicit ER on size and valence ratings. A greater tendency to use reappraisal was linked to reduced negative feelings on seeing the pictures of spiders. Greater use of suppression, however, was linked to increased size estimation of the spider stimuli.

**Discussion:**

These results highlight the role of ER in perceptual biases and offer avenues for future ER-based treatments for specific phobias.

## Introduction

Imagine that you are cleaning your cupboard when a small spider suddenly appears. While you are trying to get it out, your brother is screaming terrified, “It’s so big!!!” This situation illustrates individual differences in size estimation which is highly subjective (Stefanucci et al., 2011; Reynolds and Subasic, 2016). Many studies have attempted to determine the factors influencing the perception of size (Teachman et al., 2008; Clerkin et al., 2009; Zadra and Clore, 2011; Balcetis, 2014); fear may be one of them (Teachman et al., 2008; Leibovich et al., 2016; Youssef et al., 2016).

Several studies have shown that when individuals experience fear they overestimate the size (Leibovich et al., 2016), height (Teachman et al., 2008), and time (Bar-Haim et al., 2010) of the fear-related object. For example, spider-fearful individuals have been found to demonstrate a wide range of perceptual biases toward spider stimuli (Rachman and Cuk, 1992; Riskind et al., 1995; Teachman et al., 2008; Vasey et al., 2011; Aue et al., 2013a,b; Leibovich et al., 2016; Youssef et al., 2016; Basanovic et al., 2018). These biases can also be seen not only when participants are presented with a picture of spiders (Leibovich et al., 2016), but also when they are exposed to a living spider (Vasey et al., 2011).

Recent findings suggest that individual differences in activation of implicit (non-deliberate) emotion regulation (ER) processes, as well as in habitual use (deliberate) of ER strategies, may alter emotional reactivity. Regarding implicit ER, recent studies have indicated a role for cognitive control (Cohen et al., 2015, 2016; Cohen et al., 2016), a high-order cognitive function which enables goal-directed behavior (Gratton et al., 1988). Recruitment of cognitive control in tasks that requires the inhibition of irrelevant information was found to reduce the effects of emotionally negative stimuli on behavior (Cohen et al., 2012, 2015), physiological arousal (Cohen et al., 2015), and on emotion-related neural activity (Etkin et al., 2006, 2010; Cohen et al., 2015). For example, Cohen et al. (2015) presented an arrow flanker task (Eriksen and Eriksen, 1974), which requires ignoring distracting stimuli, prior to the presentation of negative and neutral pictures. The pictures were followed by a simple discrimination task (deciding whether a square is blue or green). Following congruent flanker stimuli (→ → → → →), the pictures led to emotional interference (i.e., longer RTs for discrimination targets that followed negative vs. neutral pictures). This effect disappeared after incongruent stimuli (→ → ← → →), which require the recruitment of cognitive control (Etkin et al., 2010; Cohen et al., 2011, 2012, 2015). These findings are in line with brain imaging data showing that activation in regions related to cognitive control (e.g., dorsolateral prefrontal cortex, anterior cingulate cortex) is associated with reduced activity in the amygdala, a region implicated in emotional processing (Etkin et al., 2006, 2010; Cohen et al., 2016). Recently, Gil et al. (2021) found that recruitment of inhibitory control (incongruent flanker stimuli) reduces the self-reported negative feeling associated with negative pictures. It is yet unknown, however, whether implicit ER can modulate fear-related responses, such as the fear associated with spider stimuli among spider-fearful individuals.

Regarding explicit ER, most studies focus on two common ER strategies: reappraisal and suppression. Reappraisal, in which a person reinterprets a situation in order to feel better about it (Gross and John, 2003), is considered an adaptive strategy. People who tend to use reappraisal more frequently experience more positive emotions and have better social interactions than those using other ER strategies (Cutuli, 2014; Dryman and Heimberg, 2018) Various lab experiments have shown that reappraisal reduces the valence of negative stimuli and the emotional arousal they elicit (Gross, 1998; Butler et al., 2003; Grisham et al., 2011; McRae et al., 2012; Buhle et al., 2014). This has also been demonstrated with fear-related stimuli such as images of snakes and spiders (Langeslag and van Strien, 2018). Similarly, reappraisal was also found to moderate subjective feelings of anxiety during a speech task among healthy individuals (Hofmann et al., 2009), as well as among individuals with math anxiety (Pizzie et al., 2020). Therefore, it seems that reappraisal may promote resilience by mitigating the relationship between stress and mental distress (for review see Riepenhausen et al., 2022).

In contrast to reappraisal, suppression is considered to be a less beneficial ER strategy (Butler et al., 2003; Gross and John, 2003). Rather than distraction, in which the person directs his or her attention away from the emotional information (Kalisch et al., 2006), when people use suppression, they inhibit their emotional responses and do not express them behaviorally (Gross, 2002). In many cases, suppression does not provide emotional relief and may increase physiological arousal (Gross and Levenson, 1993, 1997). People who tend to use suppression experience fewer positive emotions and are more likely to experience negative emotions than people using other ER strategies (Gross and John, 2003). Despite this, when combined with other strategies, suppression is associated with low symptoms of anxiety and depression among adolescents, and has been found to be effective in regulating arousal and anxiety (Keng et al., 2017; Gross and Cassidy, 2019; Yuan et al., 2020; Santos et al., 2021). These presumably contradicting findings are in line with recent theories which emphasize the importance of strategy-situation-fit (Haines et al., 2016), or emotion regulation flexibility (Kashdan and Rottenberg, 2010; Bonanno and Burton, 2013; Aldao et al., 2015). According to these two theories, well-being is a function of the “goodness of fit” between emotion regulation efforts and contextual characteristics rather than the greater widespread use of particular emotion regulation strategies (Conway and Terry, 1992; Doré et al., 2016). These ideas were contextualized following findings from ER studies showing that personal and situational factors, such as situation intensity and controllability, determine the effects of the emotion regulation attempt on the regulator’s mood (Sheppes et al., 2011, 2014; Troy et al., 2013; Scheibe et al., 2015; Wenzel et al., 2019; Shabat et al., 2021), as well as the choice or tendency to implement a specific emotion regulation strategy (Sheppes et al., 2011, 2014; Matthews et al., 2021; Shabat et al., 2021). As such, reappraisal and suppression may both be adaptive or maladaptive, depending on individual differences and situational demands (Doré et al., 2016).

Very little is known about the links between implicit and explicit ER and perceptual biases and it is still unclear whether using ER can modulate the perceptual biases associated with fear-provoking stimuli. The current study examined the links between ER and perceptual bias toward spider pictures among women highly afraid of spiders. Specifically, we tested whether implicit ER (recruitment of cognitive control) and explicit ER tendencies (habitual use of reappraisal and suppression) are associated with the size estimation and valence ratings of spider pictures.

As recruitment of cognitive control was found to reduce negative emotions (Etkin et al., 2006, 2010; Cohen et al., 2012, 2015; Gil et al., 2021), we predicted that both valence and size ratings of the spider pictures would be lower when the participants recruited cognitive control (i.e., trials in which an incongruent flanker stimulus precedes a spider picture) than when they did not (i.e., trials in which a congruent flanker stimulus precedes the picture). We also predicted that higher habitual use of reappraisal will be associated with lower size ratings and with fewer negative feelings upon watching the spider images. Furthermore, following finding showing that increased use of suppression is associated with an increase in physiological arousal (Butler et al., 2003), as well as reported negative affect (Dalgleish et al., 2009), we predicted that higher habitual use of suppression will be associated with larger size ratings and more negative feelings toward the spider pictures.

To assess whether the predicted effects are specific to the fear-related stimuli, we recruited women with high fear of spiders and compared the valence and size ratings of the spider pictures to those of butterflies and wasps. Wasps and butterflies were chosen as control stimuli based on Leibovich et al. (2016). Specifically, wasp stimuli were chosen as they are threatening for most individuals, but are not self-relevant for spider-fearful individuals. Butterflies were chosen as neutral stimuli.

## Materials and methods

### Participants

Participants were students at the University of Haifa. This study was approved by the institutional review board of the Faculty of Education, University of Haifa (No. 059/19). All methods were carried out in accordance with standard human research ethics guidelines (Declaration of Helsinki) and regulations. Written informed consent was obtained from the participants.

A power analysis using G*Power (Faul et al., 2007) revealed that a total of 34 participants are required to assess within variables interactions (i.e., Animal × Congruity) with a power >80% and *a-priori* alpha set at *p* = 0.05. Based on prior studies which showed medium to high effect size for the interaction between flanker type and picture valance (Cohen et al., 2012, 2016), we used an effect size estimate of partial *ƞ*^2^ = 0.10.

The Spider Phobia Questionnaire (SPQ; Klorman et al., 1974) was distributed on social networks and was filled out by 181 individuals. Among these individuals, 81 participants received a score of above 11 and were therefore invited to participate in the study (based on Leibovich et al., 2016). The study sample included 40 women (age range 18–35 years). Data from three women were removed due to a high error rate in the flanker task (above 15% errors, as in previous studies; Cohen et al., 2014), and data from five women were removed due to a low valence rating of the spider pictures (lower than 1 SD below the mean valence ratings of the sample). The final sample thus included 32 women.

### Stimuli

#### Flanker stimuli

Congruent and incongruent flanker stimuli were used. Congruent stimuli consisted of a row of five arrows pointing to the same direction $\left(\to \to \to \to \to \right)$ , while incongruent stimuli consisted of a row of five arrows in which the center arrow pointed in the opposite direction to the flanking arrows $\left(\to \to \leftarrow \to \to \right)$ . Participants were asked to indicate the direction of the central arrow. In incongruent stimuli, participants recruit cognitive control, while the congruent stimuli were not expected to elicit cognitive control.

#### Pictures

The animal stimuli included colored pictures of spiders, butterflies, and wasps (10 different pictures of each) in the same physical size (32 pixels), taken from Google Images.

### Questionnaires

*The Fear of spiders Questionnaire* (SPQ; Klorman et al., 1974). This is a 31-item self-report questionnaire assessing fear of spiders (e.g., “When I see a spider, I feel tense and restless”). We also added similar questions about butterflies and wasps. Cronbach alpha here for the spider-related questions was *α* = 0.89.

*Emotion Regulation Questionnaire* (ERQ; Gross and John, 2003). This questionnaire consists of 10 statements that assess two ER strategies: reappraisal (e.g., “I control my emotions by changing the way I think about the situation”) and suppression (e.g., “I keep my feelings to myself”). Participants are asked to rate whether they strongly agree or disagree with each statement on a scale from 1 to 7 (1 = strongly disagree, 7 = strongly agree). Cronbach alpha here was *α* = 0.86 for reappraisal and *α* = 0.61 for suppression.

### Procedure

Individuals who were eligible to participate in the study based on the screening questionnaire (SPQ) were invited to the lab and performed the experiment in front of a computer screen. The experiment was based on Leibovich et al. (2016) study and included a size estimation task and a valence task, and was administered *via* OpenSesame (Mathôt et al., 2012). On each trial of the task, participants were presented with a cognitive control target (congruent or incongruent flanker stimulus) that was followed by a picture of an animal (spider, wasp, or butterfly). In the first block, participants were asked to rate the perceived size of the animal appearing in the picture (size task), while in the second block they were asked to rate how bad they feel (valence task). Following this task, participants were asked to rate the unpleasantness they feel for each one of the pictures. Then, participants completed a questionnaire assessing habitual use of reappraisal and suppression, were debriefed, and thanked for their participation. The total duration of all tasks and questionnaires was about 40 min. Participants received monetary compensation or course credit for their time. All task data and materials are available at OSF: https://osf.io/vzpqc/.

#### Size estimation task

Each trial consisted of viewing a flanker stimulus followed by a size estimation task. When presented with the flanker stimulus, participants were asked to click the right mouse button if the central arrow pointed to the right, while they were asked to click the left button if the central arrow pointed to the left. Half of the trials included a congruent flanker stimulus, while half included an incongruent stimulus. The flanker stimulus was presented for 100 ms followed by a 900 ms interval, after which a picture of a spider, butterfly, or wasp was presented alongside a visual analog scale (VAS) and remained visible until a response was made but not longer than 15,000 ms. In the size estimation task, participants rated the perceived real-world size of the animal on a scale ranging from 0 to 100 with a fly on the left of the screen serving as a reference point (see Leibovich et al., 2016 for a similar design). Participants were instructed to rate the size of the animal as it appears in reality, relative to the fly presented on the left side of the line. The size task thus contained two within-subject factors: flanker type (congruent, incongruent), and animal (spiders, wasps, and butterflies). Twelve practice trials were given followed by 144 experimental trials divided into three blocks.

**Figure 1:**
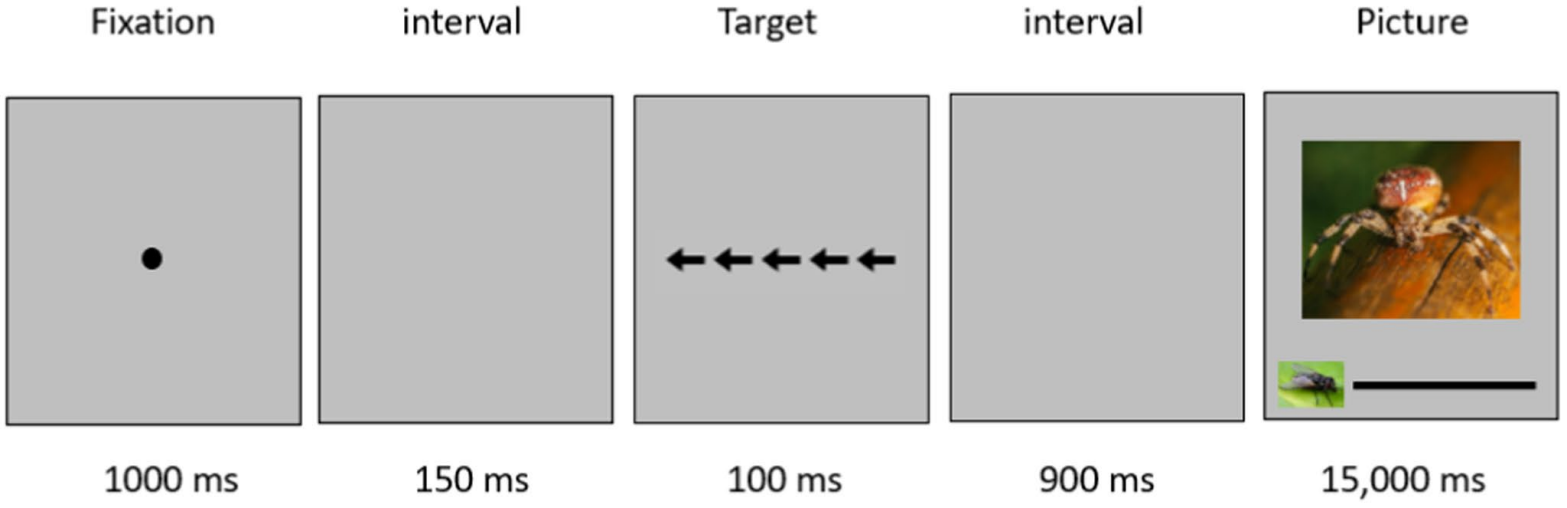
Each trial started with a fixation point shown for 1,000 ms, followed by a blank screen for 150 ms. The flanker stimulus appeared on the screen for 100 ms, followed by a 900 ms interval in which the response could be made. The animal picture along with the VAS remained until a response was made but not longer than 15,000 ms.

#### Valence task

The valence task was similar to the size task but, instead of rating the animal’s size, participants were asked to rate how bad they felt on seeing the picture. To do this they used a VAS ranging from not bad at all (left side) to very bad (right side). The valence task contained two within-subject factors: flanker type (congruent, incongruent) and animal (spiders, wasps, and butterflies). The task consisted of 144 trials divided into three blocks.

#### Unpleasantness ratings

In the third section of the experiment, to verify that the spider pictures were associated with unpleasant emotions, participants rated the degree of unpleasantness they felt when watching the pictures using a VAS ranging from not at all to very unpleasant. The task consisted of 30 trials (3 animals X 10 pictures per animal).

### Data analysis

As in previous studies (Cohen et al., 2012), trials with errors (*M* = 4.9%, *SD* = 3.1) as well as trials with extremely fast RTs (below 200 ms; *M* = 2.3%, *SD* = 6.2) in the flanker task were removed from the analysis. All analyzes were done using IBM SPSS Statistics (version 25). Repeated measures Analysis of Variance (ANOVAs) were used to examine the interactions between animal (spider, wasp, butterfly) and flanker type (congruent and incongruent). Dependent measures included size estimation and valance ratings. Unpleasantness ratings were analyzed to make sure that the spider-fearful individuals indeed rated the spider pictures as more unpleasant than wasps and butterflies. Pearson correlations between spiders’ size and valance ratings and habitual use of emotion regulation strategies (reappraisal and suppression) were also calculated.

## Results

### Unpleasantness

As expected, participants rated the spider pictures as more unpleasant than the butterfly and wasp pictures, *F*(1,32) = 8.323, *p* < 0.01, *η*^2^*_p_* = 0.212 (see Table 1 for the mean unpleasantness values of each animal).

**Table 1:** Descriptive statistics of the unpleasantness results.

	N	M	SD
Butterfly unpleasantness	32	419.346	117.721
Spider unpleasantness	32	775.054	217.404
Wasp unpleasantness	32	529.906	282.102

M, mean; SD, standard deviation.

### Congruity effect

To verify that the flanker task functioned as expected, reaction times (RT) were subjected to a repeated analysis of variance (ANOVA) with congruity as an independent factor. As expected, a main effect for congruity was found, *F*(1,32) = 107.557, *p* < 0.001, partial *ƞ*^2^ = 0.776, with slower RTs for incongruent than for congruent stimuli (*M* = 418.071 ms, *SD* = 55.559 for incongruent trials, and *M* = 378.281 ms, *SD* = 59.690 for congruent trials).

### The effect of implicit ER on size estimation

**Figure 2:**
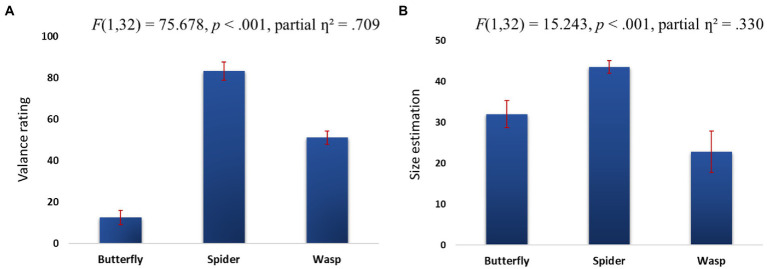
Valence ratings **(A)** and size estimation **(B)** for butterflies, spiders, and wasps. Vertical lines represent standard error.

Mean size estimation values were subjected to a repeated ANOVA with two independent variables, congruity and animal (see Table 2 for the mean animal size ratings). The results replicated those of Leibovich et al. (2016) showing an overestimation of spiders’ size compared to butterflies and wasps, *F*(1,32) = 15.243, *p* < 0.001, partial *ƞ*^2^ = 0.330. *Post-hoc* tests showed that participants rated the spiders as larger than the butterflies, *F*(1,32) = 16.156, *p* < 0.001, partial *ƞ*^2^ = 0.343, as well as the wasps, *F*(1,32) = 44.174, *p* < 0.001, partial *ƞ*^2^ = 0.588 (see Figure 2B). However, we did not find a main effect for congruity on size estimation, *F*(1,32) = 2.165, *p* = 0.151, partial *ƞ*^2^ = 0.065, nor an interaction between congruity and animal, *F*(1,32) = 1.047, *p* = 0.314, partial *ƞ*^2^ = 0.033.

**Table 2:** Descriptive statistics of the animals’ size and valence ratings.

	Butterfly	Spider	Wasp
	*M* (SD)	*M* (SD)	*M* (SD)
Size	31.3 (18.3)	45.3 (25.8)	22.3 (18.3)
Valance	11.9 (19.1)	89.8 (9.1)	56.0 (28.9)

M, mean; SD, standard deviation.

### The effect of implicit ER on valence ratings

Mean valence ratings were subjected to a repeated ANOVA with two independent variables, congruity and animal (see Table 2 for the mean animal valence ratings). There was a main effect for animal *F*(1,32) = 75.678, *p* < 0.001, partial *ƞ*^2^ = 0.709, indicating that participants rated their feelings on seeing a spider as more negative than after seeing butterflies *F*(1,32) = 476.686, *p* < 0.001, partial *ƞ*^2^ = 0.939 and wasps *F*(1,32) = 569.848, *p* < 0.001, partial *ƞ*^2^ = 0.948 (see Figure 2A). There was no main effect for congruity, *F*(1,32) = 0.604, *p* = 0.443, partial *ƞ*^2^ = 0.019, nor an interaction between congruity and animal, *F*(1,32) = 2.762, *p* = 0.107, partial *ƞ*^2^ = 0.082.

### The links between explicit ER, size, and valence ratings

**Figure 3:**
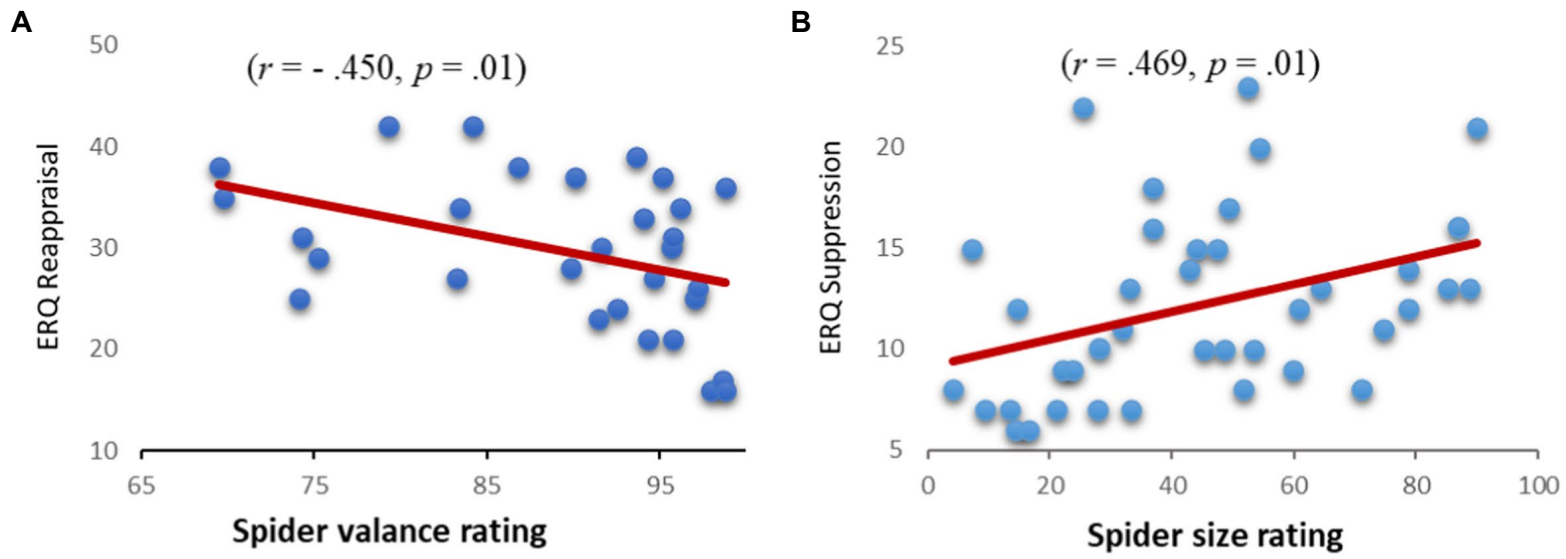
Correlation between reappraisal and spiders’ valence rating **(A)** and between suppression and the estimation of spiders’ size **(B)**.

Habitual use of reappraisal was negatively correlated with valence rating for the spiders (*r* = −0.45, *p* = 0.01), but not with the estimation of spiders’ size (*r* = 0.08, *p* = 0.65). These findings imply that participants who reported using reappraisal more frequently felt less negative when looking at the spider pictures (see Figure 3A).

Suppression was positively correlated with the estimated sizes of the spiders (*r* = 0.469, *p* < 0.001), but not with the spiders’ valence ratings (*r* = 0.08, *p* = 0.65). That is, participants who reported using suppression more frequently perceived the spiders as larger (See Figure 3B).

## Discussion

This study examined the links between emotion regulation (ER) and size and valence ratings of spider pictures by women greatly afraid of spiders. We found that women afraid of spiders showed perceptual biases toward spider stimuli, rating them as larger than butterflies and wasps (see also Rachman and Cuk, 1992; Teachman et al., 2008; Vasey et al., 2011; Leibovich et al., 2016; Basanovic et al., 2018). In contrast to our prediction, implicit ER was not associated with either size estimation or valence ratings. Higher use of reappraisal was linked to less negative feelings toward the spider pictures. Higher use of suppression was linked to the estimation of spiders as larger.

Despite previous research showing that cognitive control can serve as an implicit ER process (Etkin et al., 2006, 2010; Cohen et al., 2012, 2015), we did not observe any effect of cognitive control on size estimation and valence ratings. Specifically, size and valence ratings were similar following congruent and incongruent stimuli, indicating that they were not influenced by the recruitment of cognitive control. Several factors related to the current task design may explain these findings. First, the size estimation task required implicit emotional processing. Prior findings indicate that emotional processing of negative pictures is not influenced by cognitive control (Cohen et al., 2016), making it possible that recruitment of cognitive control also in the current task was ineffective in modulating the spiders’ size ratings. This, however, cannot explain why the flanker task did not affect the valence ratings. A second possibility for the lack of flanker effect on the size and valence ratings may be the interval from the flanker stimulus to the ratings. In previous studies, an interaction between congruity and valence was observed when a simple discrimination target was used (RTs of around 400 ms; e.g., Cohen et al., 2012, 2015). Here, participants’ average RT in the rating tasks was 1.4 s which differs significantly from the average reaction time reported in prior implicit emotion regulation tasks (around 400 ms; e.g., Cohen et al., 2012, 2015). Therefore, the relatively long time passing between the flanker stimulus and the response may have eliminated the effect of the flanker stimulus on size and valence ratings. A third account for the lack of effect of implicit ER on size and valence ratings may be related to the cognitive load that characterizes the processing of the spider stimuli. Specifically, spider phobia is not only characterized by fear of spiders, but a lot of these individuals also feel disgusted toward spiders (Mulkens et al., 1996; Olatunji et al., 2017). Disgust is known to be associated with relatively large recruitment of cognitive resources (Xu et al., 2015; Fink-Lamotte et al., 2021). As a result, cognitive abilities such as inhibitory control may be impaired or decreased (Xu et al., 2015). Regarding the current study, the recruitment of cognitive resources due to disgust may have led the implicit emotion regulation task (i.e., incongruent flankers) to be less effective in modulating the valence and size ratings of the spider pictures. Furthermore, the depletion of cognitive resources due to disgust may have caused the null effect regarding the correlation between reappraisal (which is a costly strategy) and spiders’ size. In the current study, we measured only the valence and unpleasantness associated with the spider pictures, and therefore cannot tell whether disgust played a role in the effects observed. This could be tested in future studies by asking participants to rate their level of disgust.

Finding a link between the tendency to use suppression and perceptual bias toward spider stimuli supports the idea that using suppression may be maladaptive and can even harm individuals with specific fears or phobias (Gross and Levenson, 1997; Butler et al., 2003; Asnaani et al., 2013). It is still unclear whether the tendency to use suppression is directly linked to size estimation or, alternatively, whether individuals with this tendency experience higher levels of fear and as a result see the spiders as larger. The fact that we did not find a correlation between suppression and valence ratings supports the hypothesis that suppression is not associated with a reduction of negative emotions for fear-related stimuli. Indeed, the effectiveness of suppression in reducing negative emotions is still controversial (Levitt et al., 2004; Campbell-Sills et al., 2006; Dunn et al., 2009; Kalokerinos et al., 2015; Keng et al., 2017; Katsumi et al., 2018; Yuan et al., 2020).

The link between reappraisal and low valence ratings has been widely supported in previous studies (Troy et al., 2018). These studies, however, mostly reported a reduction in negative affect following an instructed reappraisal assignment. For example, Langeslag and van Strien (2018) found that reappraisal can change emotional responses to fear-related stimuli such as images of spiders or snakes. In addition, Shurick et al. (2012) found that the reappraisal of snake and spider images resulted in a decrease in experiential and autonomic fear responses measured through electrodermal activity, which lasted 24 h after the reappraisal manipulation. There are almost no studies examining whether habitual use of reappraisal is associated with reduced affect ratings for fearful stimuli (Li and Graham, 2021). Although participants in our study were not given any instruction related to reappraisal, it is likely that those tending to use reappraisal more frequently also used this strategy during the experiment, leading to lower valence ratings.

The fact that we did not observe a link between reappraisal and size estimation contradicted our hypothesis. One explanation may be the specific characteristics of the size estimation task. As mentioned above, this task may have led to recruitment of cognitive control and spatial perception processes (Moustafa et al., 2017), known to exhaust the cognitive resources needed for reappraisal (Sheppes and Meiran, 2008; Gan et al., 2017). Furthermore, implicit processing of the spider images in this task (i.e., participants were required to process a non-emotional attribute of the stimulus) may have made the pictures more aversive (e.g., Cohen et al., 2016), making reappraisal less preferable and effective strategy (Suri et al., 2015; Scheffel et al., 2021). Therefore, although the participants were not instructed to perform reappraisal, it is possible that the automatic tendencies to use reappraisal were compromised in the size estimation task. Specifically, the size estimation task, which involved the implicit processing of spider stimuli, may have depleted the available cognitive resources required for the execution of reappraisal (Sheppes and Meiran, 2008; Hofmann et al., 2012; Sheppes et al., 2014; Suri et al., 2015; Ortner et al., 2016; Gan et al., 2017; Keng et al., 2017; Troy et al., 2018; Goldin et al., 2019). This idea fits Li and Graham (2021) study which showed that a short practice of reappraisal did not affect the size estimation of spider pictures.

The current study has several limitations. First, we manipulated implicit ER using the flanker task, which is based on recruitment of inhibitory control. Possibly using other cognitive control tasks, such as working memory or set-shifting, would have produced an effect on size estimation and/or valence ratings (Xiu et al., 2016). Second, for explicit ER we focused only on reappraisal and suppression. Other ER strategies, such as acceptance and distraction, have also been found to be effective in fear reduction (Swain et al., 2013). Third, the current study assessed only the habitual tendency to use reappraisal and suppression. We did not examine whether participants used these strategies during the task. Thus, future studies may include a question asking participants whether they have tried to implement a certain emotion regulation strategy during the experiment or instruct participants to use these strategies during the task. Namely, to examine whether suppression and reappraisal influence perceptual bias, these two strategies can be directly manipulated during a size estimation task. For example, participants may be asked to avoid showing their feelings toward the spider pictures (suppression manipulation) before rating the spiders’ size (e.g., Burns et al., 2007; Yuan et al., 2020), or to think about the pictures from a perspective of another person (reappraisal manipulation; Keng et al., 2013).

Taken together, the current study provides evidence for a link between emotion regulation and perceptual biases, as well as valence ratings. Specifically, the findings suggest that spider-fearful individuals tending to use suppression more frequently perceive spiders as larger. Furthermore, spider-fearful individuals who tend to use reappraisal more frequently experience less negative affect when seeing spider stimuli. These findings may aid the development of novel and easy-to-implement ER-based interventions designed to treat specific fears.

## Data availability statement

The datasets presented in this study can be found in online repositories. The names of the repository/repositories and accession number(s) can be found at: https://osf.io/vzpqc/.

## Ethics statement

The studies involving human participants were reviewed and approved by the questionnaires and methodology for this study was approved by the Ethics Committee of the Faculty of Education at the University of Haifa. The participants provided their written informed consent to participate in this study.

## Author contributions

YB-B, NC, and TL-R developed the experimental design. YB-B performed the data collection. YB-B performed the data analysis under the supervision of NC and TL-R. YB-B drafted the article. All authors approved the final version of the article for submission.

## Conflict of interest

The authors declare that the research was conducted in the absence of any commercial or financial relationships that could be construed as a potential conflict of interest.

## Publisher’s note

All claims expressed in this article are solely those of the authors and do not necessarily represent those of their affiliated organizations, or those of the publisher, the editors and the reviewers. Any product that may be evaluated in this article, or claim that may be made by its manufacturer, is not guaranteed or endorsed by the publisher.
